# Prebiotics: A Novel Approach to Treat Hepatocellular Carcinoma

**DOI:** 10.1155/2017/6238106

**Published:** 2017-05-10

**Authors:** Naz Fatima, Tasleem Akhtar, Nadeem Sheikh

**Affiliations:** ^1^Cell and Molecular Biology Lab, Department of Zoology, University of the Punjab, Q-A Campus, Lahore 54590, Pakistan; ^2^Cell and Applied Molecular Biology (CAMB), University of the Punjab, Q-A Campus, Lahore 54590, Pakistan

## Abstract

Hepatocellular carcinoma is one of the fatal malignancies and is considered as the third leading cause of death. Mutations, genetic modifications, dietary aflatoxins, or impairments in the regulation of oncogenic pathways may bring about liver cancer. An effective barrier against hepatotoxins is offered by gut-liver axis as a change in gut permeability and expanded translocation of lipopolysaccharides triggers the activation of Toll-like receptors which stimulate the process of hepatocarcinogenesis. Prebiotics, nondigestible oligosaccharides, have a pivotal role to play when it comes to inducing an antitumor effect. A healthy gut flora balance is imperative to downregulation of inflammatory cytokines and reducing lipopolysaccharides induced endotoxemia, thus inducing the antitumor effect.

## 1. Introduction

Hepatocellular carcinoma (HCC) ranks sixth amongst the most widely recognized malignancies and it makes up the third leading cause of cancer related deaths on a global level [1]. WHO describes HCC as a malignant tumor comprised of cells that hold a close resemblance with hepatocytes; however, their appearance is quite atypical; a plate-like union around sinusoids can be seen in almost all of them and its presence is very common somewhere in a tumor. Cirrhotic liver is deemed as the breeding ground for most of the HCCs, as nearly 80% of HCCs have begun at cirrhotic liver. Occurrence of HCC varies among the people of different geographical locations, being highest in areas like Eastern Asia and Sub-Saharan Africa. Ubiquitous chronic hepatitis B virus (HBV) infection in these areas poses a major threat to victimize the individuals with HCC [2]. Some genetic variations have been associated with human HCC, resulting in a distinctly heterogeneous profile of alterations. Genetic alterations usually entail gain and loss of chromosomal DNA, allelic loss (LOH) on some chromosomal regions, and mutations of tumor-suppressor genes and oncogenes. Some oncogenic pathways like the p53, Wnt/~-catenin pathways, and RB are impaired in HCC to a noteworthy level while others like the TGF-6 pathway are deregulated as well, but on a marginal scale. Mutation in tumor-suppressor gene p53 has been cited in almost 20% cases of HCC and interestingly, this mutation has shown a pronounced variation in the rate of mutations among tumors of different geographical whereabouts [3]. Another noticeable fact is the instance of hotspot mutation, reported in the HCCs of samples from areas with more incidence of HBV infection and elevated levels of dietary aflatoxin B1, affecting p53 at codon 249 [4].

## 2. Gut-Liver Axis

**Figure 1:**
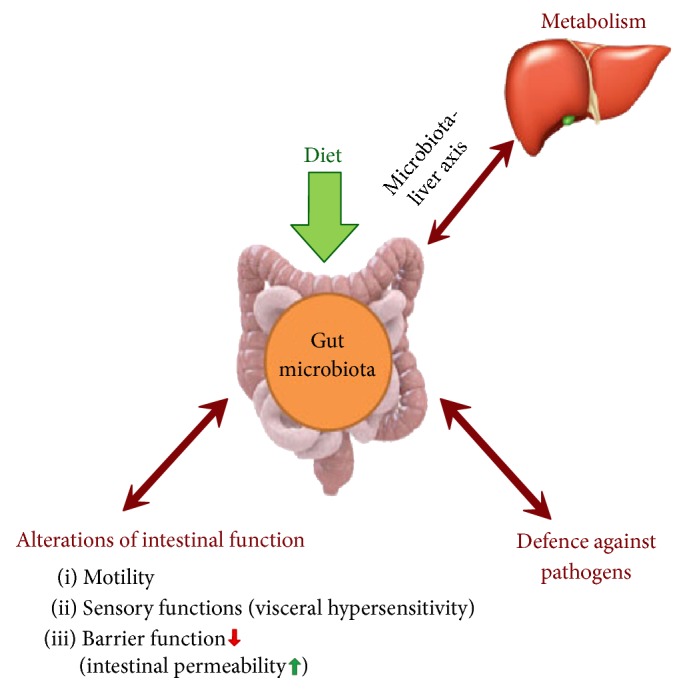
Gut-liver axis and role of microbiota.

The health of gut and liver is paramount to nutrient absorption and controlling certain chief metabolic activities. Liver is provided with a double blood supply, by the courtesy of hepatic artery and portal vein [5]. Products are gleaned from gut; lipopolysaccharide (LPS), bacterial DNA, and peptidoglycans are supplied to the liver via portal vein. The exact mechanism of gut-liver axis has been studied through a number of studies. The gastrointestinal tract is regarded high as a metabolic and immunological set-up [6] which is essential to harbor the most complex human microbial ecosystem (including gut flora) along with a vast pool of endotoxins and bacteria [7]. Studies maintain that a wide variety of intestinal microbiota, around 500–1,000 different species, are found in human intestine with an estimated population of 100 trillion microbes [8]. Intestinal mucosa makes up the major part of gut barrier along with the distribution of intestinal flora; any impairment to the gut barrier intensifies the intestinal flora which responds by an increased movement of gut microbiota across the barrier [6] and enables them to reach the liver via portal vein [9] (Figure 1).

**Figure 2:**
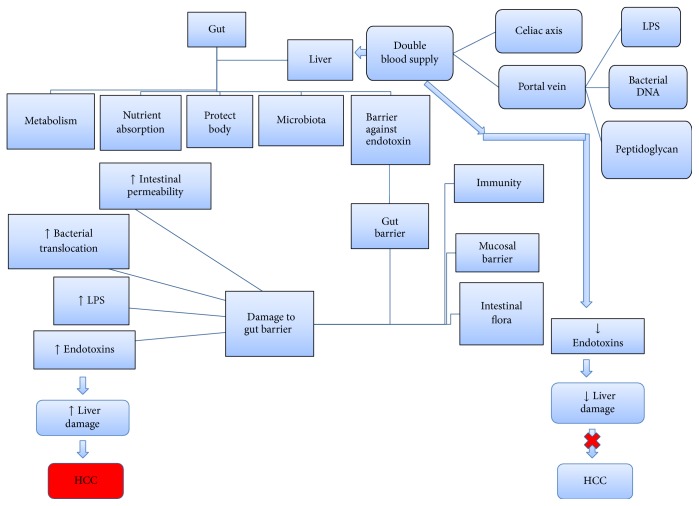
Gut-liver axis and its role in HCC.

The LPS (lipopolysaccharide) level is sensitive to overgrowth in small intestinal bacteria, changed composition of microbiota, and increased intestinal permeability [10]. Endotoxins and gut bacteria are kept at bay by a barrier offered by the gastrointestinal tract, thus protecting our body from various malignancies [11]. In addition to the above stated functions, the liver is also imperative to LPS detoxification and protection of the hepatocyte endotoxins procured by the gut [12]. A damaged liver will fail to decompose the endotoxins and expose the liver at the mercy of endotoxins to destroy the hepatocytes further [6]; hence, hepatocarcinogenesis can be promoted or this condition may serve as a target for the treatment or prophylaxis of HCC [13] (Figure 2).

## 3. Toll-Like Receptor Signaling

**Figure 3:**
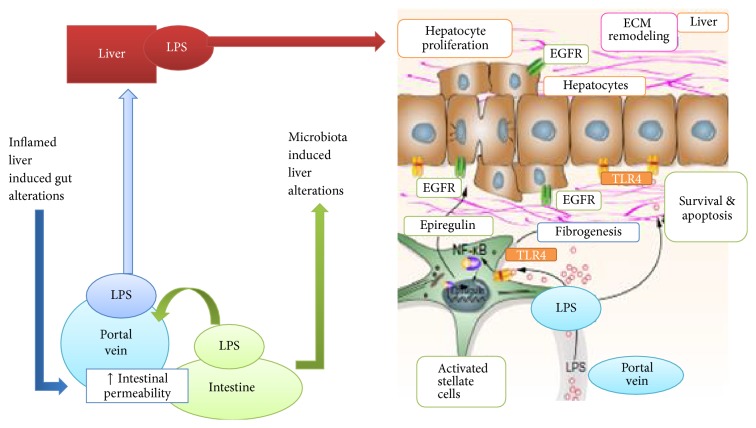
Schematic diagram showing progression of HCC by TLR4 pathway. Gut permeability alterations and LPS translocation to liver cause liver damage and activation of TLR4 signaling in HSC and hepatocytes result in ECM makeover, fibrogenesis, and exudation of EGF leading to HCC.

The gut microbial composition in the liver is often seen as a stimulator to an increased activity of liver Toll-like receptors (TLRs), a class of proteins sensitive to some structurally conserved molecules derived from the microbiota [9]. Various aspects of liver damage and chronic liver disorders like inflammation, fibrosis, and liver injury caused by a mixture of hepatotoxin carbon tetrachloride and diethylnitrosamine are susceptive of hepatocarcinogenesis when exposed to LPS-induced TLR4 signaling [14]. TLR4 signaling in liver cells (specifically in hepatic stellate cells) is believed to be associated with LPS production from the intestinal microbiota, proven by certain factors like gut sterilization, genetic TLR4 inactivation, and prolonged treatment with low-dose LPS. Epiregulin belongs to the epidermal growth factor (EGF) family and is produced as a result of activation of nuclear factor jB pathway(NF-jB) which is stimulated by TLR4 (Figure 3).

Epiregulin, along with other mediators, creates a protumorigenic medium in an already established inflammatory microenvironment, thus paving the way for HCC. TLR4 are known to promote HCC at advanced stages of liver disorders [15]. Myriads of reactions are regulated by activated NF-kB, resulting in release of several cytokines, for example, interleukin-1 and other inflammatory molecules like tumor necrosis factor-*α*, which is triggered by TLR4 activation brought about by LPS and other pathogen-associated molecular patterns (PAMPs). Furthermore, the neoplasia of lymphotoxin-induced HCC is also enhanced by the activated NF-kB in mouse [16]. By utilizing the potential of probiotics and antibiotics that cause inhibition of bacterial translocation and inhibition of TLR4 pathway by antagonist TLR4 ligands and other small molecule inhibitors of downstream signals, a delay or impairment in promotion and progression of HCC has been observed successfully (Figure 3). On the other hand, HCC chemoprevention is practiced by blocking the EGF that signals erlotinib or any other EGF receptor inhibitors as a preventive tool [17].

The activation of downstream signals is effectively prevented by a lipid A analogue, eritoran tetrasodium (E5564), which works by binding itself to internal pocket of MD-2 (coreceptor of TLR4). Inflammation induced by LPS is also significantly brought under control by E5564 that also has importance for endurance in a sepsis model [18]. Some other inhibitors like CRX-526 (antagonist ligand of TLR4) are known for inhibiting TNF-*α* production [19]. TAK-242 (resatorvid), an inhibitor of TLR-4 intracellular domain, is also regarded important because of remarkable decline in cytokine levels in mice introduced with LPS and cured with TAK-242 along with protection from LPS-induced lethality [20]. In a number of liver diseases activation of NF-kB signaling has caught the attention of the researchers, as its modulators are yet to be explored. It is also noteworthy that, due to immunosuppressive effects of TLR4 signaling, its usage has to be restricted and supervised very carefully [21].

## 4. Role of Prebiotics

**Figure 4:**
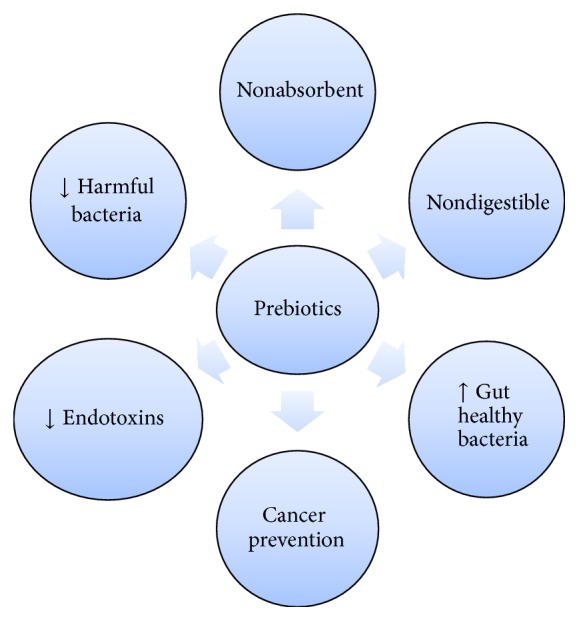
Properties of prebiotics.

HCC is advanced to a next stage as gut microbiota are an important contributive factor by virtue of gut-liver axis [22]. However, the occurrence of HCC can be warded off by bringing about changes in the type and amount of gut microbiota which poses a multitude of benefits including a healthy gut flora balance, a significant improvement in intestinal inflammation, and mucosal barrier functions, and it is also known to improve the cirrhotic condition effectively [23]. To keep a check on overgrowth of gut microbiota, various strategies can be applied involving the use of prebiotics, probiotics, and synbiotics [24]. They assist in curbing the endotoxemia by bringing about a massive decline in the population of pathogenic bacteria, achieved by tweaking the flora [24, 25] (Figure 4).

Prebiotics have a central importance in maintaining a healthy intestinal microflora balance. They are categorized under the umbrella of nonabsorbent and indigestible food ingredients like lactulose which is known to promote growth and activity of various gut friendly microbiota [26, 27]. The implications of prebiotics in preventing cancer are known widely amongst the experts [26]. Amongst the most researched prebiotics, dietary polyphenols are of key importance. They include phenolic acids, flavonoids, and lignins found in nuts, wine, tea, fruits, and vegetables. One of the important polyphenols is ellagic acid, an antioxidant having cancer-preventive properties and is metabolized by microbiota of colon into urolithins that is present in certain nuts and berries [28]. Urolithins are considered handy while downregulating COX-2-mediated inflammation which brings us to a point where we can safely state that the anticancer effects might involve a variety of pathways [29]. Pure polyphenols and polyphenol-rich foods have been shown to impart health benefits by supporting the gut friendly microbiota, along with traditional edibles as well [30]. Polyphenols are known to show chemopreventive effects in HCC [31] by immunomodulation [32]. Tea phenols have been reported to exert positive effects on gut microbial population and repress pathogenic bacteria, so they may play a role in maintaining good gastrointestinal health [33]. Moreover, tea polyphenols serve as a potent alternative for chemoprevention and treatment of HCC [34]. Furthermore, nuts are also rich in polyphenols [35], that is, ellagitannins in walnuts, raspberries and strawberries [36, 37], and proanthocyanidins (PAs) in almonds, pistachios, and hazelnuts [38]. Dietary ellagitannins show antitumor properties, though exact mechanism is still unknown [36]. PAs are condensed tannins and belong to polyphenols, found in grapes, red wine, green tea, chocolate, and other fruits and vegetables [39]. PAs alter gut microbial population and increase healthy microflora [40] and confer health benefits. Several phenolic agents in curcumin have been reported to arrest cell cycle, inhibition of proliferation, and suppression of metastasis by downregulating a number of transcription factors and cytokines in various HCC cell lines [41–45]. Another polyphenol resveratrol, naturally found in grapes [46], can also act to prevent and reduce progression of HCC [47] by suppressing metastatic invasion and cell migration in HCC [48]. Polyphenols in rice bran have also showed prebiotic effects [49]. Quercetin is a dietary flavonoid with disease prevention properties [50] works through downregulation of activated nuclear factor kappa B (NF-kB) in hepatocytes [51]. Anthocyanidin flavonoids in purple sweet potato beverage have also showed certain hepatoprotective properties [52] and, hence, may prevent progression of liver damage.

On the basis of chemical structure, the inulin-type fructans (ITF) and the galactooligosaccharides (GOS) are the two major groups of prebiotics [53]. Dietary fiber has always been considered an essential component of a healthy meal due to its positive effects on health [54]. It makes favorable conditions to support gut friendly bacteroides like* Prevotella* and* Xylanibacter*, increases the population of* Bifidobacterium*, the clostridial cluster XIVa, and* Faecalibacterium prausnitzii,* and makes conditions harsh for harmful bacteroides like* Firmicutes* and* Enterobacteriaceae* [55]. One of the gold standards to gauge the intestinal health and identify prebiotics is a sudden increase in the population of* Bifidobacteria* and* Lactobacilli*. Based on some experiments, certain fungal products have also been identified to be used as prebiotic agents in future. Some traditional Chinese therapies include using* Hirsutella sinensis* (the anamorph of* Cordyceps sinensis*),* Antrodia cinnamomea,* and* Ganoderma lucidum* as an energy booster; water mycelium extracts of this fungus have been known to reduce LPS-induced endotoxemia when used on high-fat diet mice [56] (Table 1).

Nondigestible oligosaccharides make up the majority of prebiotics identified and tested in labs so far [57, 58]. Lactulose, fructooligosaccharides (FOSs), galactooligosaccharides (GOSs), isomaltooligosaccharides (IMOs), soybean oligosaccharides (SOs), lactosucrose, glucooligosaccharides (GLOSs), xylooligosaccharides (XOSs), gentiooligosaccharides (GeOSs), mannan oligosaccharides (MOSs), arabinoxylan oligosaccharide (AXOS), chitooligosaccharide (COS), pectin-derived acidic oligosaccharides (pAOSs), agarooligosaccharide (AOS), human milk oligosaccharide (HMO), cyclodextrins, alginate-derived oligosaccharide (ADO), and xanthan-derived oligosaccharides (XDOs) are the most common nondigestible oligosaccharides which have been identified as functional in nature [59, 60]. Downregulation of low-grade inflammatory cytokines (IFN-*γ*, interleukin 1*β* [IL-1*β*]) can be brought about by triggering an increased fabrication of SCFAs in gut which can be easily achieved by incorporating diet supplemented with 10% (w/v) XOS-supplemented in the regular meals to increase* Bifidobacterium* colonies throughout the intestine to a remarkably great number. The same increase in the number of* Bifidobacteria* and* Lactobacilli* colonies can also be achieved in vitro by administering acidic oligosaccharides obtained from apple pectin which also creates an increased concentration of acetic, propionic, and lactic acid [61]. This defense barrier can be attributed to an improvised motility of inulin which offers a fair protection against cancer and bifidogenic property or to the capability of inhibiting bacterial enzymatic actions like those of *β*-glucosidase and *β*-glucuronidase [62].

**Figure 5:**
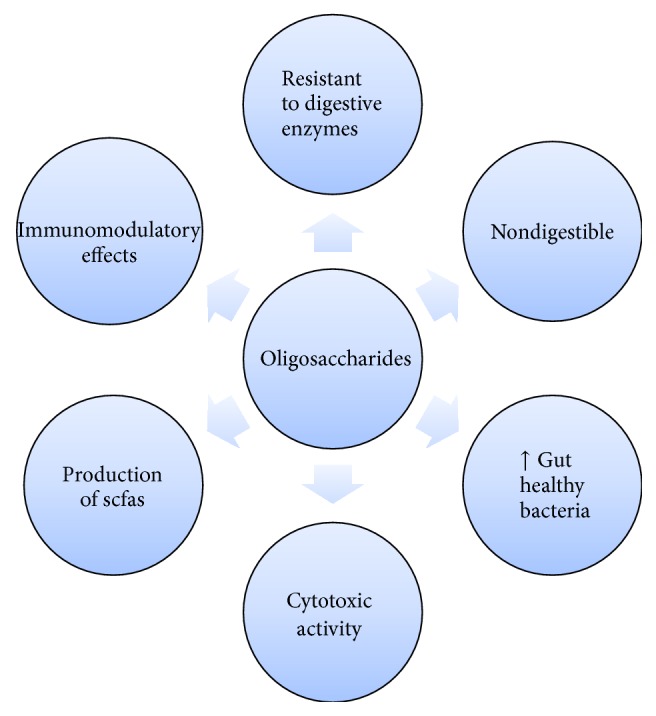
Effects of oligosaccharides.

Immune system receives a multitude of benefits, when exposed to oligosaccharides, along with successfully inhibiting cancer metastasis and carrying on certain other activities like complement activation and immunological activities [63], thus making a strong case to be administered in tumor immunotherapy. Certain structural features like glycosidic branching, typology of sugar, sulfation position, molecular weight, and degree of sulfation (DS) tend to affect the bioactivity of sulfated polysaccharides/oligosaccharides to a great deal. Antiangiogenic and antitumor activities have enormously been enhanced by chemical oversulfation of fucoidan [64, 65]. *κ*-Carrageenan oligosaccharides also showed immunomodulation effects on S180-bearing mice along with antitumor activities, extracted from* Kappaphycus striatum* [66] (Figure 5).

Cytotoxic activity of NK cells was also found to promote tumor cell elimination. NK cell's activity is observed to be enhanced by carrageenan oligosaccharides and their derivatives if administered in a dose-dependent manner. Another noteworthy finding tends to offer a comparison between potential of causing high antitumor and immunostimulatory activities, where sulfated derivatives are known to induce a significantly higher antitumor and immunostimulatory activities while the acetylated and phosphorylated derivatives have failed to impart any noticeable effect in comparison to oligosaccharides [66].

## 5. Botanical Polysaccharides Prebiotics

Uplifting the efficacy of chemotherapy has always been a challenge and botanical polysaccharides hold a special significance as they make a rich source for adjuvants, antitumor, and immunomodulating agents [67, 68]. Many polysaccharide conjugates and polysaccharides like* Ganoderma* polysaccharides [67, 69],* Astragalus* polysaccharides, lentinan, grifolan, and krestin (PSK) are known to exhibit antitumor activities by regulating the function of immune system and conducting direct actions against tumor cells [70–72]. The studies of antiproliferative effects have also been of central importance to determine the efficacy of its causative agents: ACPS-1, its major fractions ACCPS, and their effects against Hela, Skov3, HepG2, and 7721 cells in vitro. Incubation of polysaccharides together with tumor cells can halt the cell cycle and can contribute to apoptosis; some of these polysaccharides comprise a polysaccharide-peptide complex extracted from* Trametes versicolor*,* Phellinus linteus*,* Poria cocos*,* Lycium barbarum,* and* Atractylodes macrocephala* [73].

**Figure 6:**
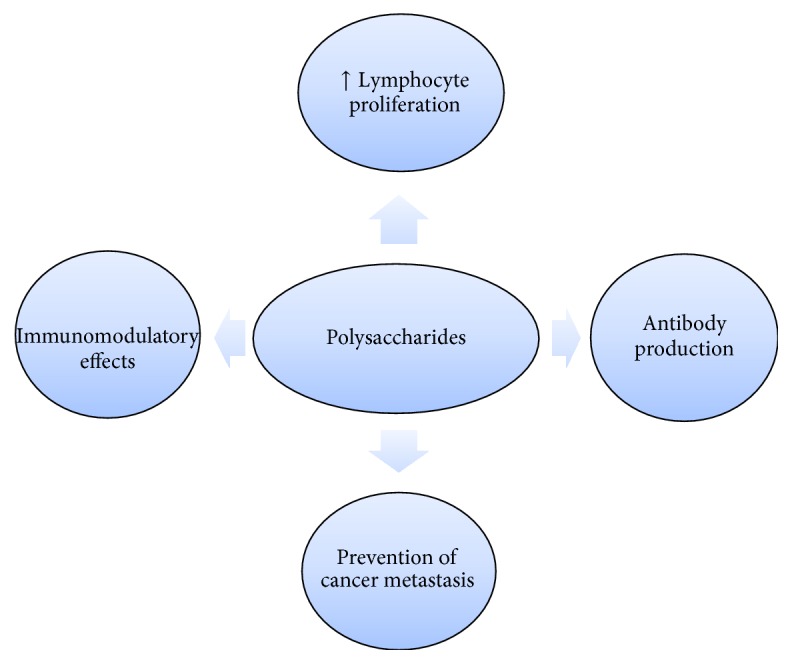
Antitumor properties of polysaccharide prebiotics.

Polysaccharides immunomodulating properties also include the enhanced proliferation of lymphocytes and antibody production, [74] as well as promoting both antitumor and antigenotoxic activities [75, 76]. Mushroom polysaccharide (sclerotia of* Pleurotus tuber-regium*) and sporoderm-broken germinating spores (SBGS) of Reishi exert a significant antitumor effect, specifically in prevention of revival or metastasis of cancerous cells. It mitigates the toxic and side effects of chemotherapy and radiotherapy in some patients [74] Moreover, polysaccharides isolated from fruiting bodies of* Pleurotus ostreatus* also have antitumor activity against Hela tumor cells [77] (Figure 6).

These polysaccharides have different chemical composition, mostly belonging to the group of b-glucans. In order to exhibit their antitumor activity, the main chains of the glucan have to be b-(1/3) linkage with additional b-(1/6) branch points [76]. The antitumor activities of Reishi polysaccharides were exhibited mainly by the branched (1/3)-b-d-glucan moiety [78]. However, the antitumor activities also depend upon several factors like solubility in water, size of the molecules, branching rate, and its form. Antitumor activity and clinical quality of polysaccharides can be enhanced by chemical modification, for instance, Smith degradation (oxidation-reduction-hydrolysis), formolysis, and carboxymethylation [76].

## 6. Fructans Prebiotics

Fructans are the most widely used prebiotics among others [79]. Oligofructose and inulin significantly alter the in vivo composition of the microbiota by stimulating the growth of* Bifidobacteria* [80]. Inulin-type fructans (ITF) are indigestible carbohydrates which decreased tumor size in hepatic and mammary tumor mouse models when administered orally [81, 82].

The gut microbiota influence progression of BaF3 cells by altering its metabolome. Due to increased gut microbiota derived metabolites which specifically target liver tissue, propionate is higher in the portal vein of rats fed with ITF and mediates a protective effect [83] and enters in liver [84]. Notably, altered gut microbiota composition or declined intake of food tends to decrease butyrate and propionate levels in cancer. Propionate having antiproliferative effect on BaF3 cells advocates that it is the most potent mediator of the ITF antitumor effect. Hence, it is speculated that uptake of propionate by liver elucidates why ITF influences progression of BaF3 cell in liver. Antitumor upshot of prebiotic nutrients may reside on propionate production by gut microbes [85].

Butyrate and other short chain fatty acids upset the cell cycle in human cancer cells by inhibiting proliferation, inducing differentiation, and cell death [86–88]. Intracellular mechanisms implicated in cell proliferation and death (initiation of caspases 3, 7 and declined histone deacetylase activity) have been widely assessed [86, 87]. Two G-protein-coupled receptors identified as receptors for SCFA are free fatty acid receptor 2 (FFA2) and FFA3, also known as GPR43 and GPR41, respectively. Most effectual endogenous agonist for free fatty acid receptors is propionate; FFA receptor 2 countenance ensues in different cell types such as intestine, adipocytes, endocrine cells, and immune cells [89, 90].

**Figure 7:**
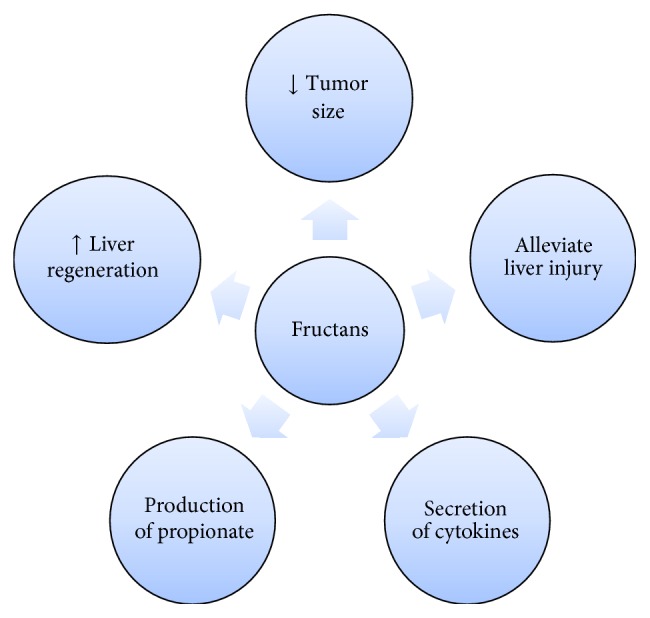
Antitumor properties of fructans prebiotics.

Administration of lactulose accelerates posthepatectomized liver regeneration in rats by inducing hydrogen, which may result from offsetting oxidative stress and inflammatory response [91]. Lactulose if orally administered could adjust the imbalance between the oxidation system and the antioxidant system of HCC patients with hepatocirrhosis and hypersplenism after interventional therapies, alleviate liver injury, and improve the antitumor immunity and prognosis [92] (Figure 7).

Cellular immunity plays an important role in tumor immunity. There is mainly the secretion of Th1 cytokines in a body having strong antitumor immunity, while the body's antitumor immunity is inhibited when there is mainly the secretion of Th2 cytokines. IFN-g and IL-4 are representative cytokines, respectively, produced by Th1 and Th2 cells. Significantly greater level of IFN-g and no difference in the level of IL-4 suggested that Th1/Th2 cell polarization evidently shifted to Th1 cells, and patient's antitumor immunity was significantly enhanced after the administration of lactulose [93]. Many other studies confirmed the positive role of lactulose in the immune defense, immune regulation [94, 95], and activation of cell mediated immune system depressed during liver cirrhosis [92].

## 7. Future Directions

Several evidences have suggested the role of prebiotics in alteration of gut microbiota and reduction of procarcinogenic factors in liver. So, in the light of antitumor properties of prebiotics, it may be suggested that modulation of gut flora by prebiotics may represent novel strategies to prevent progression of chronic liver disorders to HCC. However, further studies are still needed to confirm and clarify the possible mechanisms involved and we may hope for the development of new therapeutic strategies to prevent HCC in near future. In conclusion, this data suggests that prebiotics may prove economical and safer antitumor agents against hepatocarcinogenesis.

## Figures and Tables

**Table 1:** Showing sources and effects of different prebiotics.

Prebiotics	Sources	Effects
Polyphenols	Cocoa, tea, wine, soy products, fruits	Anticancer [30]
*Ganoderma lucidum *	Fungi	↓ LPS induced endotoxemia [56]
*Hirsutella sinensis*	Fungi	↓ LPS induced endotoxemia [56]
*Antrodia cinnamomea *	Fungi	↓ LPS induced endotoxemia [56]
Sulfated polysaccharides	Marine algae	Immune response [66]
*Kappaphycus striatum*	*κ*-Carrageenan oligosaccharides	↑ NK cell activity, antitumor activity [66]
Acidic oligosaccharides	Apple pectin	↑ *Bifidobacteria*, ↑ *Lactobacilli*, ↑ production of SCFAs [61]
