# Corrigendum to “Prebiotics: A Novel Approach to Treat Hepatocellular Carcinoma”

**DOI:** 10.1155/2017/1548919

**Published:** 2017-07-09

**Authors:** Naz Fatima, Tasleem Akhtar, Nadeem Sheikh

**Affiliations:** ^1^Cell and Molecular Biology Lab, Department of Zoology, University of the Punjab, Q-A Campus, Lahore 54590, Pakistan; ^2^Centre for Applied Molecular Biology (CAMB), University of the Punjab, Q-A Campus, Lahore 54590, Pakistan

In the article titled “Prebiotics: A Novel Approach to Treat Hepatocellular Carcinoma” [1], the second affiliation was incorrect. The correct affiliation is shown above.
